# Graphene‐Based Nanocomposites as Antibacterial, Antiviral and Antifungal Agents

**DOI:** 10.1002/adhm.202201523

**Published:** 2023-01-13

**Authors:** Seda Gungordu Er, Mohan Edirisinghe, Tanveer A. Tabish

**Affiliations:** ^1^ Department of Mechanical Engineering University College London Torrington Place London WC1E 7JE UK; ^2^ Radcliffe Department of Medicine University of Oxford Old Road Oxford OX3 7BN UK; ^3^ Department of Engineering Science University of Oxford Begbroke Science Park Oxford OX5 1PF UK

**Keywords:** antibacterial films, antifungal materials, antimicrobial films, antiviral applications, graphene, graphene oxide

## Abstract

Over the past decade, there have been many interesting studies in the scientific literature about the interaction of graphene‐based polymeric nanocomposites with microorganisms to tackle antimicrobial resistance. These studies have reported variable intensities of biocompatibility and selectivity for the nanocomposites toward a specific strain, but it is widely believed that graphene nanocomposites have antibacterial, antiviral, and antifungal activities. Such antibacterial activity is due to several mechanisms by which graphene nanocomposites can act on cells including stimulating oxidative stress; disrupting membranes due to sharp edges; greatly changing core structure mechanical strength and coarseness. However, the underlying mechanisms of graphene nanocomposites as antiviral and antifungal agents remain relatively scarce. In this review, recent advances in the synthesis, functional tailoring, and antibacterial, antiviral, and antifungal applications of graphene nanocomposites are summarized. The synthesis of graphene materials and graphene‐based polymeric nanocomposites with techniques such as pressurized gyration, electrospinning, chemical vapor deposition, and layer‐by‐layer self‐assembly is first introduced. Then, the antimicrobial mechanisms of graphene membranes are presented and demonstrated typical in vitro and in vivo studies on the use of graphene nanocomposites for antibacterial, antiviral, and antifungal applications. Finally, the review describes the biosafety, current limitations, and potential of antimicrobial graphene‐based nanocomposites.

## Graphical abstract

Over the past decade, there have been many interesting studies in the scientific literature about the interaction of graphene nanocomposites with microorganisms to tackle antimicrobial resistance. This article presents the antimicrobial mechanisms of graphene nanocomposites and demonstrates typical in‐vitro and in vivo studies on the use of graphene nanocomposites for antibacterial, antiviral, and antifungal applications.

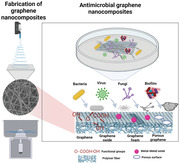

## 1 Introduction

Recently, carbon‐based materials have attracted considerable attention for the inhibition of microbial strains. Carbonaceous nanostructures can be listed as carbon nanotubes (CNTs), fullerene, diamond, graphite (Gt), and graphene.^[^ ^1^ ^]^ Carbon allotropes have played an important role in the diagnosis and treatment of wide‐ranging medical conditions including cancer, infection, brain disorders, cardiovascular diseases, etc.^[^ ^2^ ^]^ Specifically, these structures have shown excellent antimicrobial efficacies against a number of pathogens in the prevention of potential infectious diseases.^[^ ^3^ ^]^ The use of carbon‐based nanomaterials as antimicrobial agents has therefore received huge attention in recent years. However, graphene, also known as the “wonder material” among carbon allotropes, is a widely studied nanomaterial owing to its unique properties such as excellent mechanical strength, exceptionally large specific surface area, and extracellular biodegradation behaviors.^[^ ^4^, ^5^ ^]^ In the scientific literature, graphene‐based nanomaterials have a wide range of antibacterial capabilities including wrapping, membrane stress, and oxidative stress to Gram positive and Gram negative bacteria.^[^ ^1^, ^6^, ^7^ ^]^ In vivo and in vitro studies on graphene‐loaded nanocomposites, though few in number emphasized with graphene, clearly demonstrate prevention of pathogenic fungal infections.^[^ ^8^, ^9^, ^10^, ^11^ ^]^ Similarly, studies involving graphene and its derivatives have increased in recent years, especially in the development of personal protection equipment against infectious diseases caused by nonenveloped and enveloped viruses such as Ebola virus, coronavirus, norovirus.^[^ ^12^, ^13^, ^14^, ^15^, ^16^, ^17^ ^]^

Graphene is defined as a single sp^2^ hybridized carbon layer packed in a honeycomb structure.^[^ ^18^ ^]^ The structure of the graphene nanolayer is a 2D crystal of sp^2^ hybridized carbon atoms connected with a Van der Waals bond of 0.142 nm length.^[^ ^19^ ^]^ Graphene began to attract significant interest among the scientific community when Geim and Novoselov, received the Nobel Prize in 2010, for the isolation of graphene of single atomic thickness from Gt flakes using a scotch tape.^[^ ^20^ ^]^ Following the Nobel Award, several groups worldwide started investigating the unique physical, chemical, mechanical and biological features of graphene.^[^ ^21^, ^22^, ^23^ ^]^ Appearing as the thinnest and strongest material known, graphene enables research in several fields in the biomedical discipline due to its properties such as heat conductivity, electron mobility, large surface area, and mechanical strength.

**Figure 1:**
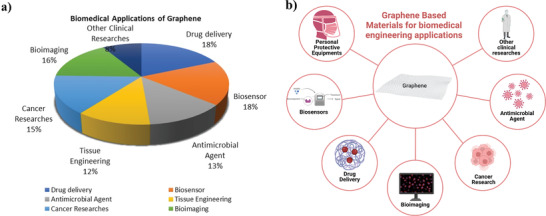
a) The distribution of graphene‐based biomedical applications b) Illustration of graphene‐based materials for use in biomedical engineering. All images within this figure are prepared in Biorender.com and have been used with permission from Biorender.

Graphene is found in a wide variety of forms which are 0D (in the form of graphene quantum dots (GQDs)), 1D (in the form of nanowires, and tubes), 2D (in the form of pristine graphene, fluorographene, graphene oxide (GO), reduced graphene oxide (rGO), porous graphene (PG), graphene nanoplatelets (GNPs), graphene nanoribbons), 3D (in the form of graphene foam, graphene aerogels). Graphene and its derivatives have extensively been explored for extended applications including^[^ ^24^ ^]^ drug delivery,^[^ ^25^, ^26^ ^]^ tissue engineering,^[^ ^26^ ^]^ sensor technologies,^[^ ^27^ ^]^ coatings for corrosion protection,^[^ ^28^ ^]^ and antimicrobial agents.^[^ ^4^, ^12^, ^14^, ^15^, ^29^ ^]^ (**Figure** **1**a,b) The fact that graphene and GO nanomaterials have a high surface area as a nanotherapeutic drug delivery platform and the high drug loading capacity of a single layer makes them attractive in this field. Graphene‐based materials can be combined with polymer nanocomposites to create dressing scaffolds that reveal good wound healing properties.^[^ ^30^, ^31^ ^]^ Due to its long‐term stability and high sensitivity, graphene has become an important field for sensing devices that are produced in biomedical engineering and are used to detect the presence of many diseases or microorganisms. The properties of large surface area and electrical conductivity enable graphene to act as an “electron wire” and enable the detection of biomolecules with its fast electron transfer feature.^[^ ^27^ ^]^ Another important research topic is the use of graphene and its derivatives in coating medical devices. It is essential to cover the surfaces of implants and medical devices with an antimicrobial agent as such healthcare‐associated infections have become a serious threat to human health.^[^ ^32^ ^]^ The superior antimicrobial performance of graphene as a coating layer ensures that the surfaces of the devices are coated against infections, polymer dressing scaffolds developed in tissue engineering can inhibit microorganisms, and personal protective equipment such as masks become a highly desirable material in preventing microorganisms. Antimicrobial agents have become crucial ingredients in the COVID‐19 pandemic, which still threatens the world today.^[^ ^33^ ^]^ Vaccine studies and antiviral drug research of this pandemic are faced with many limitations while preventing the spread of viruses with permanent mutation potential. Researchers are working on the synthesis of graphene‐based nanocomposite materials that can inhibit viruses.^[^ ^12^ ^]^

**Figure 2:**
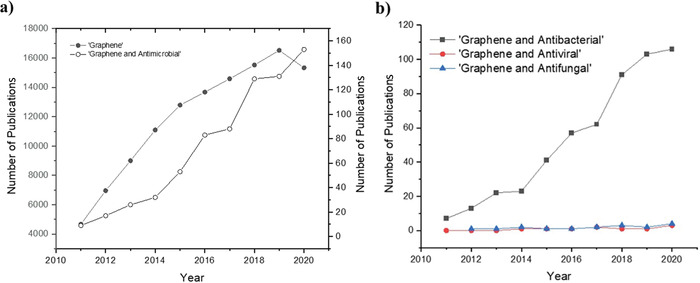
a) Number of publications for “Graphene” and “Graphene and Antimicrobial” b) Number of publications for “Graphene and Antibacterial”, “Graphene and Antiviral” and “Graphene and Antifungal”

In this review, we will discuss the synthesis, antimicrobial properties of graphene and its derivatives along with their antimicrobial mechanisms of action involving simulations. The ultimate goal is to review a range of graphene family materials, and their microbial activity in the studies that inhibit the growth of microorganisms, in bacterial, viral, or fungal strains and biofilm formations (**Figure** **2**a,b). In addition, detailed information about graphene‐based nanocomposites and physicochemical properties will also be given. Finally, in vivo and in vitro biocompatibility, challenges, and future work of graphene are discussed.

## 2 Graphene and Its Derivatives

**Figure 3:**
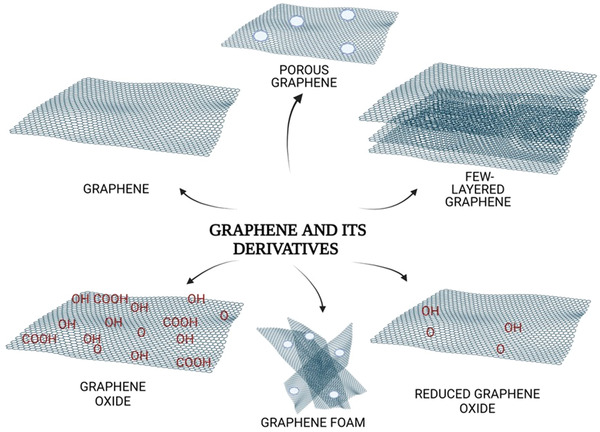
Structural illustration of graphene and its derivatives (porous graphene, graphene foam, graphene oxide, reduced graphene oxide, and few‐layered graphene). All images within this figure are prepared in Biorender.com and have been used with permission from Biorender.

After the discovery of graphene, the incorporation of graphene nanostructures into polymers has also received huge attention in order to achieve better properties in comparison to its pristine counterparts^[^ ^34^ ^]^ (**Figure** **3**).

GO and rGO nanosheets have widely been studied in the scientific literature in comparison to their counterparts.^[^ ^35^ ^]^ The functional groups onto the basal planes of GO (hydroxyl, epoxy, carbonyl, or carboxyl) provide several advantages in terms of its heterogenous electronic structure such as fluorescent in a range of wavelengths^[^ ^36^ ^]^ and its water dispersibility which is necessary for biological applications.^[^ ^37^, ^38^, ^39^ ^]^ GO and rGO form the interfaces of nano‐sized to micro‐sized molecules. In addition to being easily modified by chemical reactions, they can be used as fillers for polymeric or inorganic composites of nanoscale size.^[^ ^40^ ^]^

Graphite oxide (GtO) has a layered structure like Gt. When the layers formed are flake and the thickness of a single carbon atom, it is called GO. rGO is prepared as a result of the removal of oxygen‐containing groups from GO. GO can be synthesized cost effectively from Gt and has a hydrophilic structure and is soluble in organic solvents^[^ ^41^ ^]^ such as acetone, methanol, dimethylformamide (DMF), and water.^[^ ^39^ ^]^ The good dispersibility of GO in multiple solvents is due to the existence of functional oxygen groups on the surface of GO. GO and rGO can be functionalized with a wide variety of biomolecules (for example, antibodies, DNA and RNA) to improve their specific delivery to the site of action.^[^ ^42^ ^]^ In the study conducted to better understand the antimicrobial activity of these two graphene‐based materials, it was stated that GO showed higher antibacterial activity than rGO.^[^ ^43^ ^]^ In addition, it was observed that they showed strong antimicrobial action with direct contact membrane stress and their oxidation capacities stress.^[^ ^43^, ^44^ ^]^

Another derivative of graphene, PG, has a high specific surface area with mesoporous or microporous structure on graphene nanosheets.^[^ ^45^, ^46^ ^]^ In addition, it facilitates the passage of ions and molecules due to its high absorption property. In the study investigating PG polymer hybrid nanofibers, it was stated that these cost‐effective polymer matrices are potential candidates for ultrafiltration applications.^[^ ^47^ ^]^ Graphene foam is graphene architecture with 3D high surface area.^[^ ^48^, ^49^ ^]^ This 3D graphene with variable porous size has excellent electrical conductivity and electrochemical properties. Although it usually attracts the attention of researchers for biosensor applications, it can also be a candidate for filtration studies due to its high biocompatibility and surface area.^[^ ^48^ ^]^

The treatment of infectious diseases against continually escalating antibiotic drug resistance requires the use of antimicrobial agents that might be safe for people and do not include antibiotics. Graphene and its derivatives are among the numerous antimicrobial agents, but carbon dots are also becoming a significant antibacterial agent.^[^ ^50^, ^51^, ^52^ ^]^ Supported by its significant biocompatibility evidenced from antimicrobial testing and photodynamic inactivation of microorganisms using graphene nanostructures, along with a few adverse effects, it has been recommended as a safe bactericidal agent.^[^ ^50^, ^53^ ^]^ Fast and excellent oxidative stress of light driven carbon dots are efficient for inhibition of Gram‐negative and Gram‐positive bacteria.^[^ ^52^ ^]^

Although widely used in bioimaging and biosensing, GQDs, the 0D nanomaterial of graphene derivatives, have also been used as antibacterial agents, with high biocompatibility, aqueous solubility and stability.^[^ ^54^, ^55^, ^56^, ^57^, ^58^ ^]^ In recent years, studies conducted with GQDs nanocomposites such as silver nanoparticles (AgNps) or carbon 60 (C_60_) have increased knowledge about their antimicrobial performance.^[^ ^54^, ^57^, ^59^ ^]^ Antibacterial effects of silver–graphene quantum dots (Ag GQDs), AgNps and pristine GQDs have been compared against Gram negative and Gram positive bacteria.^[^ ^54^ ^]^ Ag GQDs were biocompatible with low cytotoxicity in mammalian cells, as well as being effective in bacterial inhibition compared to other materials. Similarly, it was observed that bacterial cells could not be inhibited by GO GQDs but by rupturing the C_60_ cage (C_60_ GQDs) inhibited only *Staphylococcus aureus* (*S. aureus)* bacteria.^[^ ^56^ ^]^ In this study, it was thought that the results were due to the connection between the GQDs source material and the bacterial cell shape.^[^ ^56^ ^]^

### 2.1 Synthesis of Graphene‐Based Nanostructures

**Figure 4:**
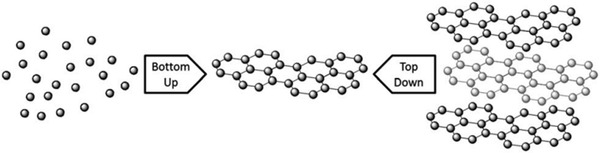
Illustration of top‐down and bottom‐up synthesis methods of graphene. Reproduced (Adapted) with permission.^[^ ^22^ ^]^ Copyright 2013, Royal Society of Chemistry.

Various methods have been reported for the synthesis of graphene‐based materials. The most popular method of obtaining graphene is the use of adhesive tape to separate thin layers of Gt and this method was first reported by Novoselov et al. in 2004.^[^ ^20^ ^]^ As illustrated in **Figure** **4**,^[^ ^22^ ^]^ the production methods of graphene can be classified into two major classes; top‐down and bottom‐up.^[^ ^20^, ^60^ ^]^

Top‐down methods include mechanical, electrochemical, oxidative, and liquid‐phase exfoliation (LPE) whereas bottom‐up routes involve chemical vapor deposition (CVD) and epitaxial growth, arc discharge, unzipping of CNTs.^[^ ^22^, ^61^, ^62^ ^]^

#### 2.1.1 Top‐Down Methods

##### Mechanical Exfoliation

This method can obtain layers mechanically, one by one by overcoming the resistance created by the van der Waals bond forces between the graphene layers in the bulk Gt.^[^ ^63^ ^]^This technique was developed by Andre Geim and Konstantin Novoselov as the first method of graphene layer derived from bulk Gt.^[^ ^20^ ^]^ With inspiration from the scotch tape method,^[^ ^20^ ^]^ another method, three‐roll milling with polymer adhesive has been successfully established for the synthesis of 1.13–1.41 nm thickness graphene layers.^[^ ^64^ ^]^ Results indicated that this presented technique can be efficacious especially for graphene–polymer nanocomposites when chosen with an appropriate polymer.^[^ ^64^ ^]^

##### Oxidative Exfoliation and Reduction

Oxidative exfoliation method has been utilized mostly in order to obtain GO.^[^ ^61^ ^]^ Following this process, rGO and pristine graphene can be obtained by reducing oxygen thermally,^[^ ^65^ ^]^ electrochemically,^[^ ^66^ ^]^ or chemically.^[^ ^67^ ^]^ At the same time, the chemical oxidation method is the most preferred method for the production of GQDs as it is simple and efficient.^[^ ^68^ ^]^ Different routes have been applied, such as; the Hummers method (using sodium nitrate (NaNO_3_) and potassium permanganate (KMnO_4)_ with the combination of sulfuric acid (H_2_SO_4_)),^[^ ^69^ ^]^ Staudenmaier method (using nitric acid (HNO_3_) and potassium chlorate (KClO_3_)) and Hofmann method (using concentrated HNO_3_ and KClO_3_).^[^ ^70^ ^]^ However, among these the most widely used approach is Hummer's method.^[^ ^61^, ^71^ ^]^ The reason is that the Hummer's method is more environmentally friendly when compared to the other methods.

##### Liquid‐Phase Exfoliation

Another approach for the fabrication of graphene is LPE which has emerged as one of the important top‐down methods. In this method, Gt disperses in a suitable liquid, then exfoliation is performed and lastly, pure graphene is obtained with the aid of high intensive ultrasound.^[^ ^72^ ^]^ Along with this method, various graphene‐based materials such as composites and films can be obtained, and have been utilized for several applications such as biosensor,^[^ ^73^ ^]^ flexible electronics.^[^ ^39^ ^]^

#### 2.1.2 Bottom‐Up Methods

##### Chemical Vapor Deposition

High quality, defect‐less graphene nanosheets with large surface area can be obtained by using this method.^[^ ^74^ ^]^ In order to grow graphene sheets, hydrocarbon gases, and other biomass components are employed on the metallic substrate at a quite high temperature, 1000 °C.^[^ ^75^ ^]^ Furthermore, this technique basically combines the carbon atoms to grow honeycomb graphene sheets. Planer few‐layered graphene was first produced in 2006 by Somani et al. using this method previously used to synthesize CNTs.^[^ ^76^ ^]^ Using this, researchers have developed a more cost‐effective and simpler production method compared to previous methods. One of the most important requirements in graphene synthesis is the controllable growth of materials.^[^ ^77^ ^]^ Approaches, where liquid metals are preferred as substrates compared to solid metals, provide an advantage for high‐quality uniform graphene growth.^[^ ^78^, ^79^, ^80^ ^]^

##### Epitaxial Growth

Another method of synthesizing graphene is to prepare it on silicon carbide (SiC) under certain conditions, at 1200–1600 °C temperature and under vacuum.^[^ ^81^ ^]^At high temperatures, Si sublimates and graphene growth occurs by collecting carbon atoms and forming sp^2^ form.^[^ ^81^ ^]^ This epitaxial graphene growth is a disadvantage of lack of homogeneity.^[^ ^82^ ^]^ Moreover, this new system has become complex due to the effect of sizes, costs, and micromachining.^[^ ^81^ ^]^ However, adjustable thickness graphene layers and high‐quality material can be obtained.^[^ ^83^, ^84^ ^]^ With the epitaxial graphene growth method, the determination of the number of graphene layers can be adjusted depending on the temperature change.^[^ ^84^ ^]^

##### Other Bottom‐Up Methods

The Arc discharge approach^[^ ^85^ ^]^ and the unzipping of CNTs^[^ ^86^ ^]^ have also recently been reported as bottom‐up methods. Whereas the arc discharge method has been previously used for the synthesis of CNTs and fullerene, more recently it has been adapted for the production of few‐layered graphene. When it comes to the unzipping of CNTs methodology, it is also useful to produce few‐layered graphene and single‐layer graphene.^[^ ^22^ ^]^ In the production of GQDs, hydrothermal or solvothermal and ultrasound‐assisted methods are applied as bottom‐up approaches. Industry constraints such as time and high heat for large‐scale production cause these methods to be less preferred.^[^ ^68^ ^]^

### 2.2 Fabrication of Graphene–Polymer Hybrid Composites

Following the overuse of antibiotics, antimicrobial drug resistance has risen in recent years. This microorganism resistance is more unlikely to spread to nanomaterials since researchers aim to design and enhance graphene nanocomposites as a potential antimicrobial method. Researchers have been drawn to graphene‐based materials in order to create new hybrid systems with improved antimicrobial properties.^[^ ^87^ ^]^ Electrically, thermally or chemically modified graphene can also be functionalized so that its physicochemical properties are useful, or different composites can be created.^[^ ^88^ ^]^ Furthermore, besides working on graphene and its derivatives as a single component, research on mixed antimicrobial agents such as metal, metal oxide, and base materials such as polymers continues.^[^ ^89^ ^]^ Graphene and derivatives obtained by various synthesis methods to obtain stronger performance with materials such as silver (Ag), zinc oxide (ZnO), titanium dioxide (TiO_2_), chitosan, and natural materials such as curcumin create synergetic effects for inhibition of bacteria, viruses, and fungi.^[^ ^14^, ^30^, ^90^, ^91^, ^92^ ^]^These hybrid components have been supported by many researchers in that they provide inhibition of various microorganisms like Gram positive and Gram negative bacteria by connecting them with binding at atomic levels through for example Van der Waals and covalent bonds.^[^ ^89^ ^]^ These graphene and metal/metal oxide composites of graphene nanosheets can be created by methods such as self‐assembly and solution blending.^[^ ^93^ ^]^ As mentioned, graphene‐based metal composites are produced for sensing, coating, and antimicrobial purposes.^[^ ^27^, ^94^ ^]^ Among these hybrid metals, Ag metal is more prominent in biomedical studies due to its biocompatible structure. In research of antimicrobial agents, Ag and GO hybrids were found to be very efficient.^[^ ^95^ ^]^ In addition, the GQDs/AgNps hybrids have been involved in research to support antimicrobial activity.^[^ ^59^ ^]^

The capacity to make graphene compatible with a variety of other chemistries, structures, and technologies is critical to expanding its use in new applications. The use of polymers to change the surface properties of graphene is a simple way to achieve this goal.^[^ ^96^ ^]^ The production of polymers polycaprolactone (PCL), polyethylene (PE), polymethylmethacrylate (PMMA), polyvinyl alcohol (PVA), polyurethane (PU) contained in these hybrid components with graphene‐based materials has been investigated with their antimicrobial properties in the production processes of diverse biomedical applications such as wound dressing and protective personal masks.^[^ ^47^, ^97^ ^]^ However, these advanced biocomposites still have various technical limitations. The distribution of graphene on the polymer, the interface interaction of graphene with the polymer, and cost‐effective and high‐quality mass production while forming this controlled structure are some of the example.^[^ ^98^ ^]^

Polymer nanocomposite solutions can be produced by solution mixing, melt blending or in situ polymerization. The solution mixing method is mostly preferred in graphene‐based polymer nanocomposites.^[^ ^99^ ^]^ This method is based on the principle of dissolving the nanofiller (like graphene and its derivatives) in a solvent and the polymer in a separate solvent and then mixing it.^[^ ^100^ ^]^ Ice bath probe and bath ultrasonication devices are used to distribute graphene‐based materials more homogeneously in the solvent.^[^ ^41^, ^101^ ^]^For GO ultrasonication, bath treatments are more preferred than probe treatment as it causes less damage to the morphology.^[^ ^102^ ^]^ For forming fibers in micro and nano sizes; electrospinning, centrifugal spinning, and pressurized gyration methods can be used.^[^ ^98^ ^]^

**Figure 5:**
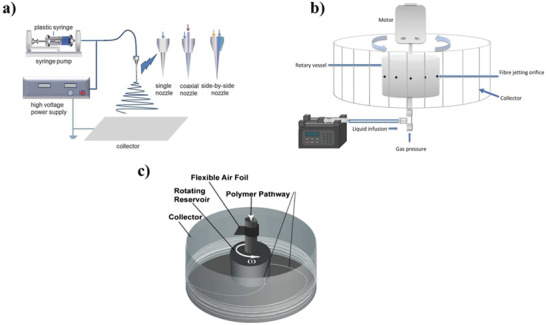
a) Basic parts of electrospinning methods such as a syringe, needle and cable, infusion pump to arrange flow rate, collector plate and power supply. Reproduced (Adapted) with permission.^[^ ^121^ ^]^ Copyright 2017, Wiley‐VCH GmbH. b) Basic parts of simultaneous pressurized gyration device such as a syringe, infusion pump, gas pressure, rotary vessel, collector, and motor. Reproduced (Adapted) with permission.^[^ ^111^ ^]^ Copyright 2017, Elsevier. c) Basic parts of centrifugal spinning method with rotating reservoir, polymer pathway, collector, flexible air foil. Reproduced (Adapted) with permission.^[^ ^108^ ^]^ Copyright 2010, American Chemical Society.

The electrospinning method, which has been encountered in science and engineering for years, has been highly preferred in polymer fiber production because it is a simple and robust technique (**Figure** **5**a). It is based on fiber production by applying a controlled electric field force to a liquid solution and overcoming the surface tension of the solution. In recent years, this method has attracted the attention of researchers for the production of nanosized fibers.^[^ ^103^ ^]^ Moreover, this technique takes advantage of the electrostatic forces which enables the fibers to have a thin diameter and large surface area.^[^ ^103^, ^104^ ^]^ In addition to being used in many fields of technology, it is a reliable method, especially in biomedical research such as sensors, enzyme immobilization, filtration, personal protection materials, drug delivery, and wound dressing.^[^ ^105^ ^]^ The production of polymeric fibers generated by the electrospinning method from the nanoscale to the low microscale is advantageous because it provides a high surface area. The high voltage applied during the method is relatively dangerous and it is not so attractive in large industrial applications involving mass production due to its low yield.^[^ ^106^ ^]^ In addition to these, this method creates difficulties during application as the low boiling temperature of the solvent used in the graphene polymer composites will cause rapid crystallization of the polymer and needle tip clogging because of the rapid solvent evaporation.^[^ ^107^ ^]^

The centrifugal spinning method, which is another polymer fiber forming method, has been suggested since a large amount of product cannot be produced with electrohydrodynamic.^[^ ^108^, ^109^ ^]^ The high‐speed rotation movement applied by the motor to the polymer solution in a cylindrical container (Figure 5c) applies centrifugal force and the surface tension of the solution is overcome, and fiber formation is generated.^[^ ^108^ ^]^ This method might be the advantageous method when used with antimicrobial agents that are sensitive to high voltage.^[^ ^110^ ^]^ It allows for obtaining smoother fibers by changing the nozzle geometry.^[^ ^108^ ^]^ One of the disadvantages of this method is that the morphology of the produced fibers cannot be controlled due to unstable applied forces.

Despite extensive research into the electrospinning method, the pressurized gyration method, has also become popular in recent fiber production. Pressurized gyration was developed by Edirisinghe and co‐workers in 2013. (Figure 5b)^[^ ^111^, ^112^, ^113^, ^114^ ^]^ By combining pressure and spinning of a solution enclosed in a perforated aluminum vessel, pressurized gyration offers a novel electric potential‐free manufacturing approach for creating polymer fibers from the micro to nanoscale in a single‐step process.^[^ ^115^, ^116^, ^117^ ^]^ It is an uncomplicated and a functional technique to achieve fibers and fibrous structures with a specific fiber size and distribution.^[^ ^115^ ^]^ With this promising method, certain limitations of other methods have been overcome by obtaining flexible fibers with high surface area and surface functions.^[^ ^118^ ^]^ In addition, a new infusion gyration approach has been developed over time, which enables the control of the flow rate of the polymer solution.^[^ ^119^ ^]^ The novel gyratory technique can also be used for the mass production of alloyed polymeric fibers.^[^ ^113^, ^120^ ^]^

## 3 Antimicrobial Activity of Graphene‐Based Materials

The prevention of some infectious diseases is seriously limited by antimicrobial drug resistance. Antimicrobial agents are essential for eradicating the drug resistance problem, which is typically caused by the overuse of antibiotics.^[^ ^122^ ^]^Additionally, the antibiofilm activity of materials incorporating graphene is a significant concern. Recent antimicrobial research has focused heavily on both the inactivation of bacteria and the suppression of the biofilm formation.^[^ ^123^ ^]^

The link between graphene physicochemical characteristics and its antimicrobial action, on the other hand, has yet to be fully determined.^[^ ^36^, ^124^, ^125^ ^]^ Among the graphene synthesis methods mentioned in the previous section, bottom‐up synthesis approaches (CVD and epitaxial growth) allow more precise production of graphene, while top‐down approaches (mechanical exfoliation, oxidative exfoliation, LPE, and other methods) can generally produce defective graphene layers.^[^ ^126^, ^127^ ^]^ These structural defects, which can occur during graphene growth, affect its performance in the area where it is used.^[^ ^128^ ^]^ However, changes in the defect‐less graphene structure can sometimes be useful as they provide new functions on binding entities and electronic properties.^[^ ^129^, ^130^, ^131^ ^]^ These defects can be easily observed with transmission electron microscopy (TEM)^[^ ^132^ ^]^ or scanning tunneling microscopy (STM).^[^ ^133^ ^]^ These intrinsic defects cause changes in the physicochemical properties and structure of graphene.^[^ ^7^, ^134^ ^]^

Physicochemical properties of graphene such as sheet size, layer number, oxidative stress‐mediation, and surface modifications substantially impact the antimicrobial activity of the material.^[^ ^135^ ^]^ Despite much effort, a number of biocidal activities and mechanisms of graphene and its derivatives based on bio‐physicochemical interfaces have not yet been fully clarified.^[^ ^136^ ^]^ In addition to these physicochemical properties; temperature, exposure time, concentration/dose, which derivatives of graphene are chosen for biocidal activity, and which metal or polymer‐based material is used with nanocomposites are also the most important factors affecting the antimicrobial activity.

### 3.1 Physicochemical Properties and Factors Affecting the Antimicrobial Activity of Graphene‐Based Materials

#### 3.1.1 Oxidative‐Stress Mediation

In most of the studies conducted on GO and rGO nanosheets, it has been observed that there are differences in the antimicrobial activities of these two materials compared to pristine graphene.^[^ ^44^, ^137^ ^]^ The significant antibacterial or antiviral effects of these materials heavily rely on time and concentration.^[^ ^12^, ^44^ ^]^ Moreover, as the lateral size decreases and the functional groups (hydroxyl, epoxy, carboxyl) increase, the toxicity impact increases thereby inducing a higher biocidal killing effect.^[^ ^138^ ^]^ Studies have also shown that even though conductive rGO has a much stronger oxidation capacity, much better antibacterial properties have been observed with GO layers have smaller lateral size dimensions.^[^ ^43^, ^139^ ^]^ Pan et al. reported that rGO nanosheets accelerate the wound healing process of oxidative stress formed by the effect of hydroxyl radical functional groups of nanocomposites formed with iron oxide nanoparticles.^[^ ^140^ ^]^ High antibacterial effect of GO‐loaded PMMA nanocomposite against *Escherichia coli (E. coli)* was observed.^[^ ^35^ ^]^ As a result of this research, it is thought that GO has a bactericidal effect due to chemical oxidative stress.

#### 3.1.2 The Number of Layers Effect

The interaction of graphene nanostructures with cells is also affected by the fact that it is single and multilayer. In the studies, it was stated that few‐layered graphene materials show a layer number dependent antimicrobial activity against *E. coli*.^[^ ^141^, ^142^ ^]^ The number of graphene layers of graphene‐based materials affects metal interaction, such as with Ag. It has been observed that the strongest interaction in 13.0 cm^−1^ for single layer with followed by 9.6 cm^−1^ for bilayer, and 9.4 cm^−1^ for trilayer graphene.^[^ ^143^ ^]^ Increasing graphene layers will result in a weaker membrane stress effect, lower dispersibility, and increased aggregation tendency, with increasing nanocomposite thickness. Therefore, it may lead to less interaction between graphene nanolayers and microorganisms.^[^ ^1^ ^]^ Similarly, in the analysis against *Streptococcus mutant* bacteria, the GNPs killing effect was observed to be much better when lower thickness and smaller size.^[^ ^144^ ^]^ The number of graphene sheets in graphene‐based polymer membranes affects the mechanical properties. In one study, the effect of graphene layer number on the mechanical properties of graphene PMMA hybrid nanocomposite was observed.^[^ ^145^ ^]^ According to the results of this research, it is observed that polymer composites formed with multi‐layer graphene have a lower Young's modulus than composites with a single layer and bilayer graphene. The reason for this is that stress formation is high between a single polymer matrix and a single graphene sheet, and as the number of layers increases, the efficiency of the stress decreases due to the slippage between them.^[^ ^146^ ^]^

#### 3.1.3 Lateral Size Dependency

Lateral dimensions of graphene are crucial to understanding its antimicrobial actions. Because the size of graphene materials (GMs) affects physicochemical interaction with microorganisms, they can penetrate the cell wall more easily. As higher lateral dimensions have more surfaces, the adsorption capability is stronger.^[^ ^7^ ^]^ The antimicrobial activities of 6 different GO sheets with lateral sizes of 0.753, 0.127, 0.065, 0.035, 0.013 and 0.010 µm^2^ were compared.^[^ ^147^ ^]^ The largest of GO sheets reduces the bacterial cell viability to ≈98% against *E. coli*, while the one with the smallest lateral area has an antibacterial activity of ≈45%. Because of their greater surface energy, GO with bigger lateral diameters is known to have better adsorption capabilities and antimicrobial action towards microbial strains.^[^ ^7^ ^]^ Therefore, it has been reported that larger dimensions of GO sheets show higher bactericidal activity against *E. Coli*.^[^ ^147^ ^]^ In addition to being size dependent, this study also emphasized that it was a time and concentration‐dependent antibacterial activity. On the other hand, it has been observed that the defects of smaller GO layers during synthesis increase microbial activity. In another study, GO sheets of varying sizes between 0, 01 and 0.65 µm^2^, a higher defect density occurs in smaller GO sheets.^[^ ^125^ ^]^ Thus, the resulting defects increase the stress on the microbial cell wall, which increases the intercellular antimicrobial properties.^[^ ^148^ ^]^ The lateral size effect of PAN polymeric graphene nanocomposite have been investigated. The large flake size (3.5 µm) of nanocomposites prepared between 0.25% and 12% concentration improves Young's modulus approximately 40% more than the small size (1 µm).^[^ ^149^ ^]^

#### 3.1.4 Surface Modification Effect

Pristine graphene might tend to agglomerate, and this reduces other molecular interactions. Because this agglomeration will reduce the contact of graphene with other molecules such as protein, lipid, DNA, and thus reduce its antimicrobial activity.^[^ ^94^, ^150^ ^]^ It was thought that the antimicrobial activity would increase with agglomerated graphene flakes. Moreover, since oxygen‐containing functional groups change the surface properties of graphene‐based materials, it is observed that the previously mentioned functional group containing materials (GO, rGO) have higher antimicrobial properties.^[^ ^151^ ^]^ As a result, it is thought that both graphene derivatives and metal composites with Ag, ZnO, TiO_2_ and graphene‐based devices embedded on polymer fiber base materials have better biocidal activities caused by surface changes.^[^ ^90^, ^152^, ^153^ ^]^

#### 3.1.5 Time and Dose Dependency

It has been observed that graphene is mostly time and concentration‐dependent in antimicrobial studies. Liu et al. mentioned that the majority of bacterial inactivation occurs within the first hour of incubation, and the rate of cell death rises steadily as the material concentration rises in their study.^[^ ^43^ ^]^ Additionally, both membrane and oxidative stress are possible causes of bacterial cytotoxicity.^[^ ^43^ ^]^ In another study, it was noted that the antiviral effects could increase with exposure for 3 and 24 h at different concentrations (0, 2, 4, or 8 wt% of GNPs or GO nanosheets).^[^ ^12^ ^]^

### 3.2 Main Antimicrobial Mechanisms and Simulations of GMs

**Figure 6:**
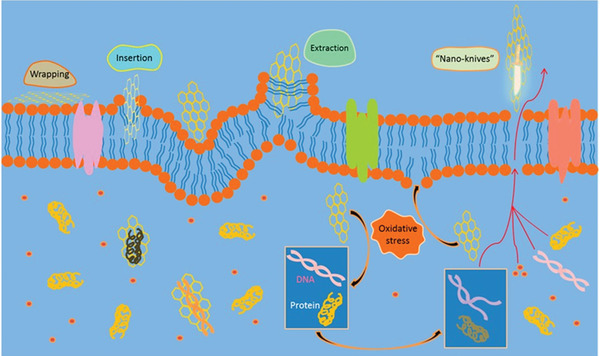
The antimicrobial mechanisms hypothesis of graphene and its derivatives on a cellular structure. These are wrapping, insertion/extraction, nano‐knives and oxidative stress. Reproduced (Adapted) with permission.^[^ ^7^ ^]^ Copyright 2010, American Chemical Society.

It is very important to understand the interaction between graphene‐based materials and microorganisms such as bacteria, viruses, and fungi. Although graphene is generally believed to inactivate bacteria in the contact‐killing mode, its variable intrinsic properties make it difficult to define these mechanisms precisely.^[^ ^136^ ^]^ Various hypotheses of these mechanisms are included in studies such as nano knives, wrapping, and oxidative stress^[^ ^1^, ^7^ ^]^ (**Figure** **6**).

The nanoknives mechanism mode, which is also called the “insertion mode,” is the mode that defines the easy entry of the microorganism into the membrane and killing the cell, since the edges of graphene‐based materials are sharp like knives.^[^ ^7^ ^]^ It has been reported in studies that graphene‐based materials cause cell death by the flowing of DNA or RNA cell cytoplasm due to their sharp edge properties.^[^ ^153^, ^154^ ^]^ In this mechanism, graphene layer thickness and hydrophilicity may affect the degradation of membrane integrity of single‐celled microorganisms.^[^ ^155^, ^156^ ^]^ In addition, the angle of orientation between the graphene‐based material and the microorganism also affects the antimicrobial efficiency.^[^ ^157^ ^]^ This mechanism will probably not work when the GMs with sharp edges lie parallel on the microorganism membrane layer. However, during parallel behavior, the pores on the graphene surface may damage the phospholipid structure of the microorganism and cause cell death.^[^ ^158^ ^]^

Simulation studies of cell membrane interaction and lipid extraction indicated that pristine graphene and GO layers may penetrate the microbial surface and achieve cell death.^[^ ^159^ ^]^ Simulated experiments were conducted to demonstrate cell membrane penetration.^[^ ^156^, ^159^, ^160^ ^]^According to the findings of Tu et al. Van der Walls bond and hydrophobic interactions may enhance the antimicrobial impact. In addition, damage not only to the cell membrane but also to the phospholipids simulated the death of the cell. In another simulation, the mechanisms of pristine graphene and GO nanomaterials were examined, while pristine graphene quickly inserted into the membrane, GO always remained at the interface during the simulation.^[^ ^160^ ^]^ It can be because of the hydrophobic contact between the lipid molecules and GO. Li et al. reported that the interaction of graphene and few‐layered graphene with cell surface and lipid using molecular dynamics and in vitro cell imaging.^[^ ^161^ ^]^ According to this simulation, cell uptake can begin by spontaneously penetrating the membrane locally at corners and asperities, then spreading spontaneously along the graphene edge to complete the penetration.

Another of the main mechanisms of graphene can be specified as the “oxidative stress” mode. Oxidative stress formed inside the cell due to the reactive oxygen species (ROS) causes degeneration of the cell membrane and triggers cell necrosis.^[^ ^162^ ^]^ It is generally known that when the generation and removal of ROS are out of balance, cells can no longer withstand the accumulated oxidative damage using their intrinsic repair systems. Additionally, it appears that GMs can directly cause oxidative stress without using ROS. Moreover, certain metal oxides (like ZnO) included in nanocomposites contribute to boost the formation of ROS in composite materials of graphene with the help of light.^[^ ^92^, ^95^, ^163^ ^]^ These photocatalytic materials increase the biocidal effect of polymer graphene hybrids. Graphene nanocomposites, which are composed of some polymers such as quaternized chitosan,^[^ ^164^, ^165^ ^]^ provide inhibition of microorganisms as it increases ROS production when an electric potential is applied. The antimicrobial mechanism in graphene–Ag nanocomposites is that Ag^+^ ions destroy the cell using the contact‐killing mode.^[^ ^8^, ^89^, ^166^ ^]^

Finally, the “wrapping” mode is defined as a hypothesis arising from the unique flexibility of graphene and its atomic thickness. As is known, cells continue their vital activities by getting enough nutrients and throwing out waste. A single layer of graphene, which does not contain pores, has a structure that does not allow the passage of many nutrients, including oxygen and carbon dioxide.^[^ ^167^ ^]^ Even if certain molecules are still permeable from its defects, microbial proliferation is prevented because it will form a highly impermeable encapsulation.^[^ ^125^, ^147^ ^]^ In this process, exposure time is very important for antimicrobial efficiency. It is stated that graphene‐based materials, with their physical and mechanical properties, surround the microorganism environmentally and insulate them in order not to maintain their vitality.^[^ ^1^ ^]^ These extraordinary properties of graphene and its ability to encapsulate microorganisms have a significant antimicrobial effect. Although attempts are made to define antibacterial mechanisms in the literature, the mechanisms related to antiviral graphene‐based materials are still unclear and require more research to help clarification.

### 3.3 Antibacterial Applications of Graphene‐Derived Materials

**Figure 7:**
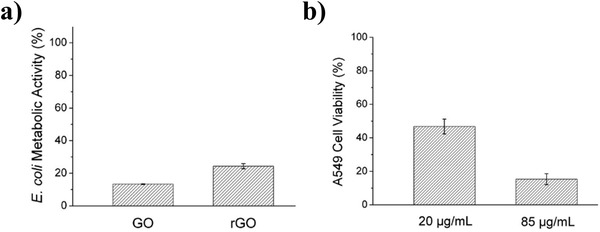
Antibacterial activity for GO and rGO. a) *E. coli* metabolic activity for GO and rGO b)  A549 Cell viability at 20 µg mL^‐1^ and 85 µg mL^‐1^. Reproduced (Adapted) with permission.^[^ ^168^ ^]^ Copyright 2010, American Chemical Society.

The study conducted by Hu and co‐workers is the first study involving graphene as an antibacterial agent.^[^ ^168^ ^]^ In this study, the bactericidal activity of GO and rGO nanosheets against *E. Coli* bacteria was reported for the first time. This report highlights the cost‐effective and mass production advantages of GO, in addition to the antimicrobial effects of graphene‐based antibacterial scenarios. In addition, in the observations made with TEM, it was observed that GO nanosheets increased cytotoxicity by about 20% to 50% (from 20 to 85 µg mL^‐1^) for A549 mammalian cells^[^ ^168^ ^]^ (**Figure** **7**a,b). More importantly, GO exhibited higher antibacterial properties compared to rGO, while exhibiting less biocompatible properties. It was observed that the metabolic activity of *E. coli* bacteria lost up to 98.5% for the highly concentrated GO nanomaterial.

**Figure 8:**
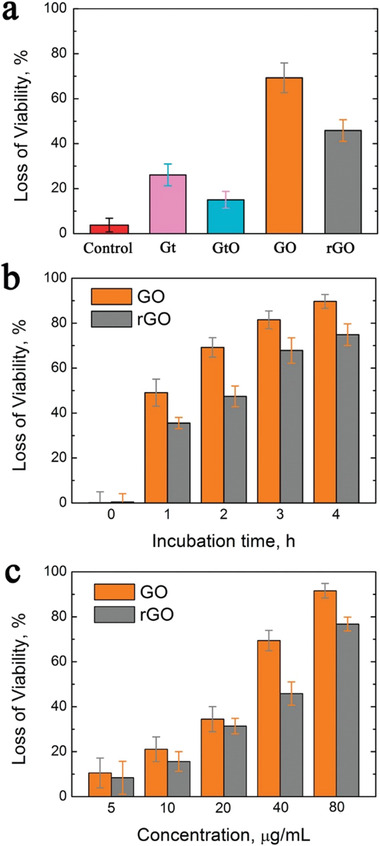
Antibacterial activity for Gt, GtO, GO, and rGO. a) Cell viability measurement of Gt, GtO, GO, rGO b) Loss of viability during incubation time (0,1, 2, 3, 4 h) for GO and rGO c) Loss of viability with different the concentrations (5, 10, 20, 40, 80 µg mL^‐1^) of GO and rGO. Reproduced (Adapted) with permission.^[^ ^43^ ^]^ Copyright 2011, American Chemical Society.

In another study, when GO and rGO nanomaterials were compared, it is seen that the antibacterial activity of GO is higher.^[^ ^43^ ^]^ (**Figure** **8**a–c) However, it can be stated that the agglomerate susceptibility of graphene‐based materials will decrease the efficiency of killing microorganisms.

Tu et al. reported measurements of antimicrobial effect against *E. coli* bacteria.^[^ ^169^ ^]^ There are two different mechanisms by which *E. Coli* bacteria are inhibited in the study. While one of them is the mechanism mentioned before as nano knives, another is the destructive extraction of lipid molecules in the cell membrane It has been reported that both mentioned mechanisms create severe membrane stress, and significantly reduce the viability of *E. coli* bacteria. According to the results of this study, antibacterial activity varies depending on the lateral size and concentration (90.9% antimicrobial activity at 500 nm dimensions). However, since surface modification can change the edge properties of graphene and derivatives, it can reduce antibacterial activity.

It has been observed that nanocomposites formed by combinations of graphene and its derivatives with metals or metal oxides have antibacterial activity. Among these, the efficiency of the hybrid of rGO nanosheets with ZnO metal oxide against *E. coli* bacteria was observed by TEM.^[^ ^163^ ^]^According to the results of this study, rGO and rGO–ZnO metal oxide both have antibacterial activity. However, rGO–ZnO showed a 50% higher bactericidal effect than rGO (inhibition zones were 28 ± 0.7 mm and 18 ± 0.5 mm, respectively.) Moreover, this synergetic effect of rGO and ZnO can be useful for biomedical research since their toxicity to mammalian cells is low.

In biomedical applications, various antimicrobial agents such as AgNps, TiO_2_, ZnO, and Tellurium nanoparticles are used in the prevention of microbial infections.^[^ ^30^, ^170^ ^]^ In these processes, graphene and its derivatives combined in a nanocomposite play an important role in destroying bacteria since it shows an effective biocidal effect. AgNps are often preferred in medical products due to their antimicrobial properties. However, safety tests for the environment and humans are still being carried out. It has been noted in the studies that the cytotoxic effects of AgNps can change as size‐dependent.^[^ ^171^ ^]^ This feature shows that the dimensions should not be ignored in the use of biomedicines. At the same time, the synthesis method of AgNps also affects cellular toxicity.^[^ ^172^ ^]^ It has been stated that biosynthesis showed higher cytotoxicity compared with chemically synthesized using AgNps. It has been emphasized that the cytotoxicity effects of AgNps may be the effect of oxidative stress related to ROS.^[^ ^173^ ^]^ Interestingly, AgNps coated with polyvinylpyrrolidone (PVP) did not show cytotoxicity.^[^ ^174^ ^]^ It has been reported that the higher cytotoxicity of GO‐AgNps nanocomposite, which is a concern in biomedical applications, is higher when compared to its pristine counterparts.^[^ ^175^ ^]^ In the study, it was observed that it is dose‐dependent, size‐dependent and temperature‐dependent using GO‐AgNps nanosheets.^[^ ^166^ ^]^Likewise, it was recorded that lower concentration, lower temperature and smaller size AgNps have effective antimicrobial activity for *E. coli* bacteria and *Pseudomonas aeruginosa* bacteria.^[^ ^166^ ^]^

Tellurium, another material whose antibacterial effect has been observed, has also been embedded on polymeric fiber meshes (PMMA as a polymer) using pressurized gyration and experiments were conducted against *E. coli* bacteria.^[^ ^170^ ^]^ Here, bacterial growth tended to be dose dependent. The highest 1.16 log reduction was recorded with 4wt% tellurium. However, studies are continuing to eliminate the uncertainties about toxicity.^[^ ^170^ ^]^ Moreover, information on the antimicrobial activity of tellurium and graphene hybrids is limited in the literature.

**Figure 9:**
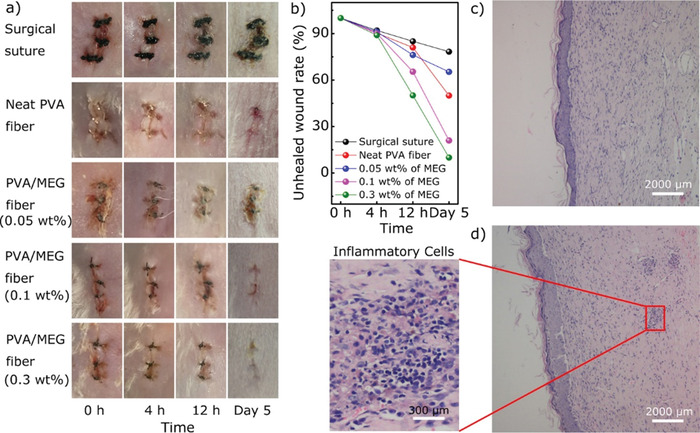
Wound healing process of mice model. a) Wounds representative after surgery with different concentrations of mechanically exfoliated graphene (MEG)(0.05 wt%, 0.1 wt%, 0.3wt%) in different time scales (0 h, 4 h, 12 h and 5 d). b) Unhealed wound rate in five groups in 5 d. c) Nanocomposite fiber d) Inflammatory cells representative. Reproduced (Adapted) with permission.^[^ ^176^ ^]^ Copyright 2018, American Chemical Society.

In vivo antibacterial analysis of graphene polymer hybrids is also included in the literature. PVA and mechanically exfoliated graphene nanocomposite fiber used in biomedical applications have been evaluated for wound healing.^[^ ^176^ ^]^In this study on mice models, it has been reported that 0.3 wt% of mechanically exfoliated graphene surgery suture heals in 5 days and has good antibacterial effects with low toxicity (**Figure** **9**). When comparison with the results of a different study, the novel nanomaterial created with the Chitosan / PVA / GO combination developed for tissue engineering prevented in vitro and in vivo bacterial growth. The inhibition of all bacterial growth was achieved with 0.75 wt% of GO.^[^ ^91^ ^]^

Researchers have demonstrated that ultrafiltration graphene‐incorporated membrane with modified surfaces can remove bacteria as well as inhibit them. Membranes with high surface area GO containing magnetic nanocomposite were studied for the removal of bacteria.^[^ ^177^, ^178^ ^]^ Zhan et al. conducted bacteria removal tests with magnetic graphene nanocomposite.^[^ ^178^ ^]^ Using Fe_3_O_4_ as a hybrid nanomaterial, the researchers studied the removal of various bacteria such as *S. aureus, E. coli*. The results showed approximately 93.09% removal efficiency for graphene nanocomposite for *E. coli*, while it was 54.97% without graphene. In the study conducted for bacteria in the real water sample, it was found to have high removal (94.8%). In another study of graphene nanocomposite antibacterial removal, poly(N‐vinylcarbazole) (PVK) was used to modify graphene and GO membrane filter.^[^ ^153^ ^]^ In the bactericidal experiments performed with *E. coli* and *Bacillus subtilis*, it was observed that the presence of graphene improved antimicrobial activity. In the analyzed results, *E. coli* and *Bacillus subtilis* removal efficiency were measured as 3 and 4 log, respectively. These results highlight that graphene is a potential candidate for bacteria removal. Bacteria removal studies have been mostly successful in wastewater treatments and results elucidate its effectiveness for biomedical filtration equipment.

### 3.4 Antiviral Applications of Graphene‐Derived Materials

**Figure 10:**
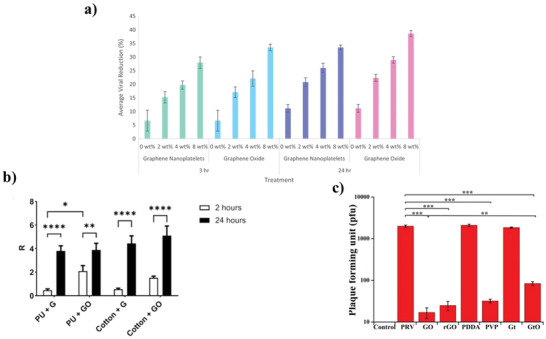
a) Antiviral activity of PMMA fibers loaded with 0, 2, 4, or 8 wt% of GNPs or GO nanosheets during 3 and 24 h. Reproduced (Adapted) with permission.^[^ ^12^ ^]^ Copyright 2020, Europe PMC. b) Graphene (G) and GO on the PU and cotton antiviral efficiency during exposure time (2–24 h). Reproduced (Adapted) with permission. Copyright 2021, Elsevier. c) Plaque‐reduction assay of PRV in the absence or presence of GO, rGO, GO‐PDDA, GO‐PVP, Gt, and GtO. Reproduced (Adapted) with permission.^[^ ^15^ ^]^ Copyright 2015, American Chemical Society.

There is extensive research on the antibacterial activity of graphene and its derivatives. However, studies on antiviral effects are still limited. In the study conducted by Ye et al., GO antiviral activity was evaluated against *Pseudorabies virus* (*PRV*) and *Porcine epidemic diarrhea virus* (*PEDV*).^[^ ^15^ ^]^ According to the results of this study, reported to be dependent on time and concentration, higher antiviral activity was demonstrated when comparing single‐layer GO and rGO with Gt and GtO, respectively. Additionally, when creating a composite with PVP (a nonionic polymer), GO had significant antiviral action, but these results did not show similar effects when conjugated with poly(diallyldimethylammonium)chloride (PDDA, a cationic polymer) (**Figure** **10**c). Interestingly, GO has a significant antiviral effect even in low concentrations.

Previous studies reported that the multifunctional nanomaterial B‐cyclodextrin (CD) functionalized GO and curcumin developed by Yang et al. has a strong antiviral effect at a concentration ranging from 0.3 to 10 µg mL^−1^ for 24, 48, and 72 h.^[^ ^16^ ^]^ In addition to the fact that these results are dose‐dependent, it is stated that the antiviral effect of this new functional nanomaterial against *RSV* infection shows very favorable biocompatibility to host cells.

The activity of GO–AgNps nanocomposite, against Porcine reproductive and respiratory (PRRVS) has been evaluated.^[^ ^179^ ^]^ Although GO has antimicrobial activity, AgNPs hybrid is higher than AgNPs and GO materials individually and prevents virus entry. The results showing 59.2% inhibition efficiency are not exceptional though.^[^ ^179^ ^]^ When GO–Ag nanoparticles were examined against enveloped infectious bursal disease virus (IBDV) and non‐enveloped Feline coronavirus (FCOV), the inhibition of infection was found to be 23% for IBDV for 25% FCOV, while it was reported that only the GO nanomaterial had an efficiency against FCOV as 16% and did not show any antiviral activity for IBDV.^[^ ^14^ ^]^

In the study by Matharu et al., both the pure antiviral effect of carbonaceous materials such as GNPs and GO and the antiviral effect when loaded onto the polymer fiber were observed against *T4 bacteriophage* with 3 h and 24 h exposure time.^[^ ^12^ ^]^ (Figure 10a) Physical and chemical interactions are thought to be involved in both materials' antiviral mechanisms, with direct nanoparticle contact being the most common route of action.^[^ ^12^ ^]^Antiviral activity on treatment GNPs and GO polymer was measured as 39%. As a result, direct contact with pure nanomaterials leads to much higher inhibition results.

The antiviral activity of GO and GO–Ag against enveloped and nonenveloped viruses was investigated comparatively in the previous study.^[^ ^14^ ^]^ In fact, although the antiviral activity of GO and GO–Ag has not been studied much, it is known that GO and GO‐Ag have significant antibacterial effects.^[^ ^43^, ^181^ ^]^ Since GO sheets have different antiviral efficacy against enveloped and non‐enveloped viruses, it is possible to suppose that there is physical or chemical contact between them and coronavirus envelope, resulting in lower virus infection.^[^ ^14^ ^]^ Without an envelope, the antiviral activity would be completely dependent on Ag particles, however, GO sheets can enhance Ag particle antiviral activity by promoting uniform dispersion and the production of spherical particles free of aggregations. Furthermore, immobilizing AgNPs on GO sheets lowered cytotoxicity, which is generally produced by free AgNPs. According to the result of this study antiviral action was shown in GO sheets containing AgNps against both enveloped and nonenveloped viruses, but GO sheets alone could only suppress the infection of enveloped viruses at noncytotoxic doses.^[^ ^14^ ^]^

Deokar et al. conducted research on the photothermal antiviral properties of graphene to destroy *Herpes simplex virus* type 1 (HSV‐1).^[^ ^182^ ^]^In this study, the antiviral effects of magnetic nanoparticles (MNPs) and sulfonated magnetic nanoparticles functionalized with reduced graphene oxide (SMRGO) are discussed. SMRGO has been shown to be an antiviral agent that is both effective and fast (99.99% in 7 min).^[^ ^182^ ^]^ The ease with which graphene may be functionalized with MNPs allows for the aggregation of trapped viruses at a specific location using an external magnet, promoting successful photothermal therapy. MNPs and SMRGO were acting against virions in the same way.^[^ ^182^ ^]^ These results are promising in that the photothermal effect of graphene may also be effective in other virus infections.

Researchers have questioned whether graphene and GO nanomaterials are effective for the COVID‐19 pandemic.^[^ ^180^, ^183^, ^184^ ^]^ De Maio et al. reported that given the particular suppression of live Sars‐CoV‐2 particles, the data provided in this study promote the continued development of graphene or GO incorporation into face mask materials (Figure 10b).^[^ ^180^ ^]^ The initial objective is to create a feasible and useful material that is resistant to thermal, mechanical, and other stresses and can be used in masks and personal protective equipment (PPE) for broad usage. Face masks, which are now widely recommended in areas where COVID‐19 is present, can help to minimize viral transmission and protect global health.^[^ ^180^ ^]^

### 3.5 Antifungal Applications of Graphene‐Derived Materials

Antimicrobial studies with graphene showed that antibacterial studies were more intense compared to antiviral or antifungal studies. In the research conducted by Li et al., the antifungal effect of 0.02, 0.2, 1, 5, 25% concentration GO and GO–AgNps sheets against *Candida albicans* and *Candida tropical* was observed for clinical applications such as nosocomial infections, candidal vaginitis, and wound.^[^ ^8^ ^]^ While GO–AgNps provides increased antifungal activity, interestingly, it has been noted that pure GO nanomaterial is not effective in killing fungi. In another study, *Aspergillus  niger, Aspergillus oryzae (A. oryzae)* and *Fusarium oxysporum (F. oxysporum)* fungi were used and show that rGO is antifungally efficient.^[^ ^10^ ^]^ However, the limitation of this study is that rGO is toxic to harmful fungi as well as to useful fungi such as *A. oryzae*.

The antifungal effect of GO and Borneol (GOB) hybrid has also been evaluated.^[^ ^9^ ^]^ It was recorded that GOB, which is a novel material, is a biocompatible material in addition to having very substantial antifungal activity. On the other hand, it is known to have antifungal effects other than the antibacterial activity, mentioned previously on the graphene–TiO_2_ nanocomposite.^[^ ^90^ ^]^ Moreover, this material has both self‐cleaning properties and negligible toxicity in delivery antimicrobial. Likewise, in another antimicrobial investigation of GO with *E. coli and S. aureus, Candida albicans*, due to disruption to the membrane of microorganisms, a dispersion of GO at a concentration of 29 mg mL^‐1^ has been found to have an antibacterial and antifungal effect.^[^ ^185^ ^]^ Following 2 h of action with the GO solution, the microorganism cell membrane is broken and dies, resulting in the greatest antimicrobial impact. According to the results, bacteria and fungus both have time‐dependent sensitivity with respect to GO.^[^ ^185^ ^]^

**Figure 11:**
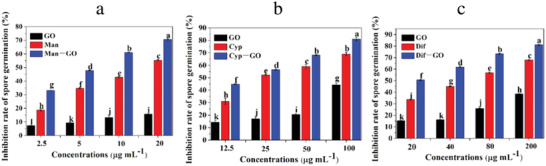
Histograms graph of inhibition rate of FG with a) GO‐Man, b) GO‐Cyp and c) GO‐Dif. Reproduced (Adapted) with permission under the terms of the CC‐BY license.^[^^11]^ Copyright 2021, the Authors. Published by MDPI.

GO is also found to be an efficient antifungal agent against plant pathogens in agricultural science. Researchers have observed that GO with a combination of fungicides (Mancozeb (Man), Cyproconazol (Cyp), and Difenoconazole (Dif)) shows substantial inhibitory effects against *Fusarium graminearum* (FG). The ratios of GO and fungicides have changed from 1:9 to 9:1. In the results of this study pure fungicides and pure GO inhibitory efficiency were obtained lower than synergetic GO‐fungicides; GO‐Man, GO‐Cyp, and GO‐Dif could significantly reduce vitality level of FG around 61%, 75% and 50%, respectively (**Figure** **11**a–c).^[^ ^11^ ^]^ The antibacterial, antiviral and antifungal activities of graphene and its derivatives are summarized in **Table** **1** below.

**Table 1:** Summary of antimicrobial applications

Graphene type/ composite	Antimicrobial Name	Effectivity and highlights		Mechanism of action	Cytotoxicity	Refs.
Graphene oxide	*E. coli*	98,5%	Cost‐effective and mass production advantages	Oxidative stress or physical disruption	Relatively biocompatible, Mild cytotoxicity	[168]
Graphene oxide	*E. coli*	91.6 ± 3.2%	Time and concentration dependent antibacterial activity	Membrane and oxidation stress	–	[43]
Graphene oxide/ /PDDA and PVP	*PRV*, *PEDV*	2 log reduction	Time and concentration dependent antiviral activity	Sharp edge and negative charge or polymer wrap	Around 80%‐100% cell viability with GO at different concentrations	[15]
Graphene oxide and graphene nanoplatelets/PMMA	*T4 bacteriophage*	39%	The pristine GO has much more effectivity than composite	Physical and chemical interactions /oxidative stress	–	[12]
Reduced graphene oxide	*E. coli*	<98,5%	GO shows higher antibacterial activity when compared to rGO	Oxidative stress or physical disruption	Lower cytotoxicity when compared to GO	[168]
Reduced graphene oxide	*E. coli*	74.9%	Particle size and oxidation capacity can influence the antibacterial activity. Also, rGO can demonstrate stronger antibacterial activity with direct interactions with bacteria	Membrane and oxidation stress	–	[43]
Graphene oxide/AgNPs	*E. coli* *P. aeruginosa*, *S. aureus*, *Candida* *Albicans*	–	Shows time and dose dependent antimicrobial activity. Lower temperature is significant for effectivity. Pristine GO is more effective	Oxidative stress	GO–AgNPs are more toxic with pristine GO due to synergetic effect between GO and AgNPs	[166]
Graphene oxide/AgNPs	*FCoV* *IBDV*	59,2%	The study shows that this composite is more effective than GO and AgNps	Oxidative stress and insertion/ extraction	GO and GO–Ag did not show cytotoxicity at lower concentration	[14]
Graphene oxide / AgNPs	*Candida albicans Candida tropicalis*	–	GO/AgNps do not show any antifungal activity. Carbon nanoscrolls /AgNps is effective for fungi inhibition	Oxidative stress	Concentration dependent cytotoxicity was observed	[8]
Reduced graphene oxide/ ZnO	*E. coli*	–	Comparing rGO with rGO/ZnO, rGO/ZnO shows better effectivity than rGO. Cytotoxicity is low for mammalian cells	Disruption of the bacterial cell	rGO is less cytotoxic	[163]
Graphene/ PVA	*E. coli*, *S. aureus*,	97,6%	Mechanically exfoliated graphene nanocomposite has low toxicity and antibacterial activity	Physical damage/ sharp edges and oxidative stress	Low cytotoxicity	[176]
GO/ chitosan/ PVA	*Bacillus cereus* *S. aureus* *Salmonella spp*. *E. coli*,	–	This new nanocomposite is effective in preventing bacterial growth	These findings point to the films' potential for tissue engineering and cell regeneration when made using GO/ chitosan/ PVA nanocomposites	–	[91]
GO/ curcumin	*RSV*	–	High biocompatibility and effective for viral inhibition	RSV is directly inactivated, the virus's adhesion to host cells is inhibited, and viral replication is disrupted	A high level of biocompatibility towards the host cells	[16]
GO/ borneol	*Mucor racemosus (M. racemosus)*	–	GOB has great antifungal activity comparing with GO and rGO	Needle like nanostructure of GO and its surface stereochemistry	GOB is non‐cytotoxic and can be utilized for a variety of purposes	[9]

### 3.6 Antibiofilm Activity of Graphene‐Derived Materials

Biofilms are known as solid barriers that surround bacteria and prevent the penetration of external antibacterial reagents. Biofilms are structures that are challenging to eradicate with different antimicrobial treatments, like antibiotic‐resistant bacteria. Graphene and its derivatives can prevent or remove biofilm formations as well as affecting independently living microorganisms. Jang et al. examined the removal of biofilm of Gram positive *staphylococcus epidermidis* and Gram negative *P. aeruginosa* bacteria with a composite of GO and AgNPs at different concentrations (4‐250 µg mL^‐1^).^[^ ^186^ ^]^ They tested this by covering the microfluidic channels with the biofilm and reported that both increased antibacterial activities. According to the results bacterial biofilm growth was inhibited at 31 µg mL^‐1^ with no toxicity. A similar study tested the effect of GO against *S. aureus, P. aeruginosa* bacteria, and *Candida albicans* in chronic wounds and for biofilm prevention,^[^ ^123^ ^]^ and significant restraint of biofilm formation has been reported in this study.

Zhao et al. examined the ability of polyethyleneimine (PEI) and GO–Ag nanocomposites to inhibit the growth of bacteria, fungi, and the biofilm formation at concentrations of 5 and 10 µg mL^‐1^.^[^ ^187^ ^]^ The nanocomposite, which prevents about 99% of bacteria and fungi, thus preventing the biofilm, was promising in combating biofilm in biomedical applications. In addition, it has been noted that PEI polymer hybrid results are more effective than plain GO–Ag for preventing agglomeration. Metal oxides (AgO, ZnO, and nickel oxide (NiO)) and rGO nanocomposites were investigated for many different bacteria (*E. coli, P. aeruginosa, S. aureus, Bacillus subtilis*) and fungi (*Candida albicans, Cryptococcus neoformans, A. niger, Aspergillus terreus, Aspergillus flavus, and Aspergillus fumigatus*).^[^ ^188^ ^]^ The active concentration of rGO nanocomposites ranged from 0.97 to 10.0 µg mL^‐1^ for antimicrobial activity and findings showed that the NiO nanohybrid demonstrated a relatively stronger antimicrobial effect compared to other metal oxides and was successful in inhibiting biofilm formation.

## 4 Biocompatibility of Graphene‐Based Materials

Graphene nanoparticles (GNPs) can be loaded into a variety of polymeric composites to improve their mechanical, thermal, and electrical properties. While these polymer hybrid GMs are used in antimicrobial applications, it is expected not to decrease the biocompatibility but to support the reduction and inhibition of microbial growth.^[^ ^189^ ^]^ This is a critical issue because infections are widespread in biomaterial implantation procedures, wound healing patches, and other biomedical applications to protect patients. Microbial viability is reported to be reduced following exposure to graphene and its compounds in nanocomposites in numerous research. The physicochemical features of graphene, such as oxidative stress, sharp edges, layer number, and layer thickness, all contribute to this decline. In addition, the concentration of the material and the exposure time can increase the antimicrobial activity as well as negatively affect biocompatibility.

Various in vivo and in vitro investigations have been used to try to understand the cellular toxicity rate of graphene‐based compounds. GNPs were used to treat male rats for a month while Kanakia et al. monitored the organ and physiological damage.^[^ ^190^ ^]^ Dextran was used to cover GNPs at different doses between 1 and 500 mg kg^‐1^. There has not been any discernible harm to hematological, cardiovascular, or respiratory structures in the range of the highest acceptable dose, which is 50–125 mg kg^‐1^. The conclusions are crucial for human studies. In vivo biocompatibility studies were carried out with 3D graphene foam with Cyprinus carpio.^[^ ^48^ ^]^ In the control group, four different doses of graphene foam, 5, 10, and 15 mg L^‐1^, were observed for 7 d for their effects on liver, kidney, and heart. While the findings did not show any critical toxicity at the highest dose, indicate changes in some biochemical blood parameters.

The cytotoxic effects of graphene and single‐wall carbon nanotubes (SWCNT) on neural pheochromocytoma‐derived PC12 Cells were investigated by Zhang et al., and it was shown that these effects vary depending on concentration and shape.^[^ ^191^ ^]^ Hep G2 cells were used to test the cytotoxicity of GO and carboxyl graphene (CXYG) nanoplatelets. The findings reveal that cytotoxicity is dosage and time‐dependent for both graphene derivatives, with no cytotoxicity observed at lower doses of roughly 4 g mL^‐1^.^[^ ^192^ ^]^ The biocompatibility of PCL and well‐dispersed GO composites was tested using a rat fibroblast cell line (L‐929). The scaffolds demonstrated no toxicity and strong cell adhesion across all surfaces, indicating acceptable acute in vitro biocompatibility and justifying future development for in vivo research.^[^ ^193^ ^]^ Wang et al. observed in vivo biocompatibility with different doses of GO injected through the tail veins of mice.^[^ ^194^ ^]^ While no significant toxicity was observed in mice at 0.1 mg and 0.25 mg concentrations, it was observed that mice became physically weaker at higher doses (0.4 mg). It has been reported that about half of the mice suffered mortality after the first 7 d.

In vitro toxicity tests of GQDs for photodynamic treatment were performed using NIH3T3 mouse fibroblast cells and 16HBE14o‐ human bronchial epithelial cells. In addition, adult male Sprague‐Dawley rats were utilized to assess the toxicity of GQDs in vivo. Tabish et al. investigated the in vivo toxicity of these GQDs in rats. After four weeks of high‐dosage administration, they found high biocompatibility and moderate toxicity, both of which are important for future therapeutic applications.^[^ ^195^ ^]^

rGO was studied to better understand chemical‐biological interactions in SKMES‐1 and A549 lung cancer cells, and the results suggest that toxicity is dosage‐dependent.^[^ ^196^ ^]^ The effects of graphene nanopores on these lung cancer cells and rats were also examined. The findings suggest that there is limited bioavailability on biological tissues and cancer cells.^[^ ^197^ ^]^ In another study, the biocompatibility of rGO and poly(vinylidene fluoride) (PVDF) composites was investigated. Human umbilical vein endothelial cells (HUVECs) disseminated and proliferated more substantially on the composite membranes, indicating increased cell adhesion and proliferation as well as improved membrane biocompatibility.^[^ ^198^ ^]^

In table below (**Table** **2**), the toxicity results of graphene nanocomposites at different concentrations are summarized in most cases and mostly dose‐dependent toxicity is observed.

**Table 2:** Graphene‐based nanocomposites with different concentrations and their biocompatibility

Graphene derivatives	Concentrations	Application	Forming method	Material	Notes	Refs.
GO	1, 1.5, 3 wt%	L‐929 mouse fibroblast cells	Electrospinning	12% PCU/DMF: THF	1% GO maximum cell viability, %3 GO minimum.	[199]
GO	0.1 0.3 0.5 1 wt%	L‐929 mouse fibroblast cells	Electrospinning	GMGO or AuGO /PVA:water solution (7 wt%, 10 wt%, 13 wt%, 16.67 wt%)	A negligible cellular toxicity and hence promising biocompatibility	[200]
Graphene	5, 10, 15, 20 wt%	L‐929 mouse fibroblast cells Tissue‐engineered nerve grafts	Electrospinning	Gr/SF (silk‐fibroin) composite	The addition of Graphene had no obvious cytotoxic effect. Cell viability with Gr/SF was higher than with pure SF membranes	[201]
GO	0.5, 1, 2 wt%	Wound dressing	Electrospinning	12 wt% Polyethylene oxide (PEO) /chloroform: Ethanol DP scaffold (80:20 wt%)	Nontoxic and does not induce any acute inflammatory responses	[202]
GO	0.25, 0.5, 1.0 wt%	L‐929 mouse fibroblast cells Tissue engineering‐wound dressing	Electrospinning	PVA/GO: acetic acid/distilled water (1: 1, v/v)	0.25% GO is the most suitable scaffold for cell viability	[203]

The toxicity and biocompatibility of graphene‐containing nanomaterials have been investigated in many studies to prevent potential health hazards. However, in vitro and in vivo related studies are still under development and should be analyzed in detail.^[^ ^204^ ^]^ Graphene‐based materials do not show toxicity up to a certain concentration, however it can be dangerous at high doses. Controlled functionalization of graphene nanomaterial and appropriate adjustment of the administered dose are important for reducing toxicity. Encapsulation of graphene‐based materials with polymer hybrids may reduce potential toxicity.

The compatibility of the equipment used in biomedical engineering with the human body is one of the most important factors. In other words, it is expected that the material taken into the body will not be toxic to human cells as long as it encounters it, including by air. In addition, the fact that medical equipment or devices are long‐lasting does not develop antimicrobial resistance, have high biocidal activity, and have low cost are among the limitations of biomedical products. The vast majority of research on graphene and its derivatives is done to meet these requirements, therefore, the role of graphene‐based nanomaterials in the development of medical devices and biomaterials is enormous.

GO is soluble in water but it can aggregate when dispersed in saline environments such as phosphate‐buffered saline.^[^ ^205^ ^]^ Since aggregation causes bead formation in fiber mats, this limitation should be considered for polymer solutions prepared for nanofiber formation. When producing graphene‐based materials, it is rare for all of layer to be of equal size.^[^ ^206^ ^]^ These various layers can affect both the biocompatibility findings and lead to inconclusive results in vivo and in vitro, chemical impurities are also of critical importance for biological evaluations.

Although the processes and antibacterial applications of graphene and its derivatives nanocomposites are widely understood, the mechanisms underlying their antiviral and antifungal properties are still unclear. One of the key components in the creation of an antimicrobial agent is understanding the processes and physiochemical properties that affect biocompatibility. In order to combat drug‐resistant microorganisms and biofilm formations, it is also crucial to comprehend the interaction between the microbial membrane and graphene nanomaterial. Therefore, future studies on graphene nanocomposites as antimicrobial agents should be more focused on the microbial inhibition mechanisms.

In 2020, COVID‐19, which is a viral pandemic that threatens the world and still continues to be a danger for many countries overshadowed the research described in this review. As this is the case, researchers are curious about the role of graphene and its derivatives in preventing COVID‐19 due to its antimicrobial properties.^[^ ^183^, ^184^, ^207^, ^208^ ^]^ Personal protection equipment have been one of the most important areas in dealing with COVID‐19, especially face mask use has been supported by the World Health Organization, to prevent the spread of the virus among people. With the emergence of the virus and the threat to the world, the antiviral activities of the protective face masks, have accelerated this research. It has become essential to protect people working in important work areas, especially healthcare workers and the role of graphene in biomedical engineering research has taken an even more crucial role.

The antibacterial effect of GO and its synergetic effect with AgNps have been extensively studied in the literature. However, synergetic effects with conventional molecules such as nitrogen, boron, and sulfur should be investigated instead of traditional antimicrobial agents.^[^ ^209^ ^]^ In addition, graphene derivatives such as other PG, graphene foam, GQDs should be examined in antimicrobial studies with different synergetic antimicrobial agents in future work.

## 5 Concluding Remarks

The biomedical research of graphene and its derivatives has been extensively studied by many researchers in recent years. Although many studies such as wound healing, controlled drug release, sensor technologies and cancer treatments have frequently been reviewed in the literature, studies that highlight the antimicrobial properties of graphene‐based materials have not been extensively covered. Especially in the COVID‐19 pandemic process, the search for antiviral agents has increased rapidly and graphene and its derivatives are key material here in various morphologies. However, concerns about the level of cytotoxicity, antimicrobial efficiency, and unexplained modes of action in mammalian cells with respect to graphene and its derivatives are often questioned. Understanding the bio‐physicochemical interactions of graphene is crucial to the development of different nanocomposites, providing new research possibilities. In this article, antimicrobial graphene has been reviewed in detail from its derivatives and nanohybrid synthesis to its applications. Moreover, the effects of physicochemical properties on antimicrobial performance as well as results of time, temperature and concentration‐dependent results are included in this review by comparing the antimicrobial efficacies of different derivatives of graphene. The biocompatibility of the materials used in antimicrobial applications has also been discussed. Like many studies reviewed in this study, graphene nanocomposites are promising healthcare components for antimicrobial coatings, personal protection equipment such as face masks, wound dressings and prevention of antibiotic drug resistance.

## Conflict of Interest

The authors declare no conflict of interest.

## Author Contributions

S.G.E. performed conceptualization, resources, and visualization, and wrote original draft. M.E. and T.A.T. involved in conceptualization, overall supervision, reviewed and edited the manuscript.
